# Training of primary care physicians enhances performance of mobile teledermatology^☆^^☆☆^

**DOI:** 10.1016/j.abd.2020.07.017

**Published:** 2021-05-21

**Authors:** Cesare Massone, Sanja Javor, Ilaria Amato, Giovanni Biondo, Alexandra Maria Giovanna Brunasso, Rainer Hofmann-Wellenhof

**Affiliations:** a Dermatology Unit, Galliera Hospital, Genoa, Italy; b University of Palermo, Palermo, Italy; c Istituto Clinico Sant’Ambrogio, Gruppo Ospedaliero San Donato, Milan, Italy; d Department of Dermatology, Medical University of Graz, Graz, Austria

Dear Editor,

Several studies over the past 20-years have proven that Teledermatology (TD) is cost-effective and leads to appropriate diagnosis and treatment decisions.^1^ Store-And-Forward Teledermatology (SAF-TD) appears to be cost-effective when used as a triage.^1^

However, it has been also reported that TD-based screening of skin cancer is associated with an increasing rate of consultations concerning benign skin conditions (i.e. seborrheic keratosis) that do not require a dermatological intervention.^2^, ^3^ Moreover, in some cases image quality is not good enough to perform TD.^3^, ^4^ If requests regarding benign skin condition increase constantly or images are not judgeable especially when resources for TD are limited (i.e. low number of teleconsultants), performances of TD may decrease.

The authors of the present study performed a TD monocentric pilot study with General Practitioners (GPs) that received specific training in dermatology and TD (ethic committee approval #114REG2017 09.11.2017). A mobile-phone application with a specific website log-in and password protected were developed (https://galliera.telederm.it; e-derm-consult GmbH, Graz, Austria).

Fourteen GPs received a 6-h pre-study personal interactive training course on: a) skin cancers, psoriasis, acne; b) how to acquire standardized digital images; c) how to access the applications and how to generate an anonymous request. In their daily practice during February 2017 and February 2019 the participating GPs enrolled 231 patients (M: F = 124:107; age 15–99 years; mean age: 64-years) with lesions suspicious for skin cancer, severe psoriasis or severe acne. All patients signed informed consent. GPs acquired 1–3 digital photographs with the mobile-phone application and generated an anonymous request. A teleconsultant answered the requests reporting diagnosis and image quality on the CRF (primary endpoint) and arranged an appointment for FTF evaluation for each patient. FTF (that represented the diagnostic gold standard) was performed by a different dermatologist and not the teleconsultant. At FTF the second primary endpoint, namely the appropriateness of the requests (defined as a consultation that requires a dermatological intervention) was evaluated.

Two-hundred and fifty-four requests were generated (23 patients had two requests). FTF diagnoses were 44.9% Non-Melanoma Skin Cancers (NMSC) (19.3% basal cell carcinoma [49/254], 15.4% squamous cell carcinoma [39/254], 10.2% actinic keratoses [26/254]); 22.8% seborrheic keratosis (58/254); 17.7% atypical melanocytic nevi (45/254); 5.5% melanoma (14/254); 3.1% angiomas (8/254); 2.8% severe acne (7/254); 1.6% severe psoriasis (4/254); 0.4% lymphomatoid papulosis (1/254); 0.4% neurofibroma (1/254); 0.4% verruca vulgaris (1/254); 0.4% perioral dermatitis (1/254).

The number of teleconsultations needed to identify cancer (pick-up rate) was 1:2.03 for NMSC and 1:16.5 for melanoma.

Image quality was optimal in 224/254 cases (96.9%), good in 3/254 (1.3%), sufficient in 2/254 (0.9%), and insufficient in 2/254 (0.9%). TD diagnosis and FTF examination showed an agreement of 0.92 with Kappa Cohen.

The authors observed that 72.9% of requests resulted appropriated (Fig. 1), requiring a dermatological intervention like i.e., surgery for skin cancers, topical therapies for actinic keratosis, dermoscopy evaluation for atypical moles or melanoma or systemic therapies for severe psoriasis or acne. Contrarily, in a previous skin cancer triage TD-study, it was observed that 82% of teleconsultations requested by GPs were considered not appropriate due to the benign nature, and intervention was not needed.^3^ Also, only 77% of clinical images were of excellent quality, 19% of moderate and 4% of low image quality.^3^ Consequently, only 26 skin cancers in 690 patients (2.6%) were diagnosed.^3^ Similarly, also Moreno-Ramirez in a cross-sectional study over 7-years on 34,553-patients reported that 39.5% of teleconsultations regarded benign skin conditions (23.8% seborrheic keratosis, 15.7% other benign skin condition) and reported a pick-up rate for malignant lesions of 1:8.6 (1:9.6 for NMSC and 1:78.8 for melanoma), with an increasing trendline for seborrheic keratosis over 7 years.^2^

**Figure 1:**
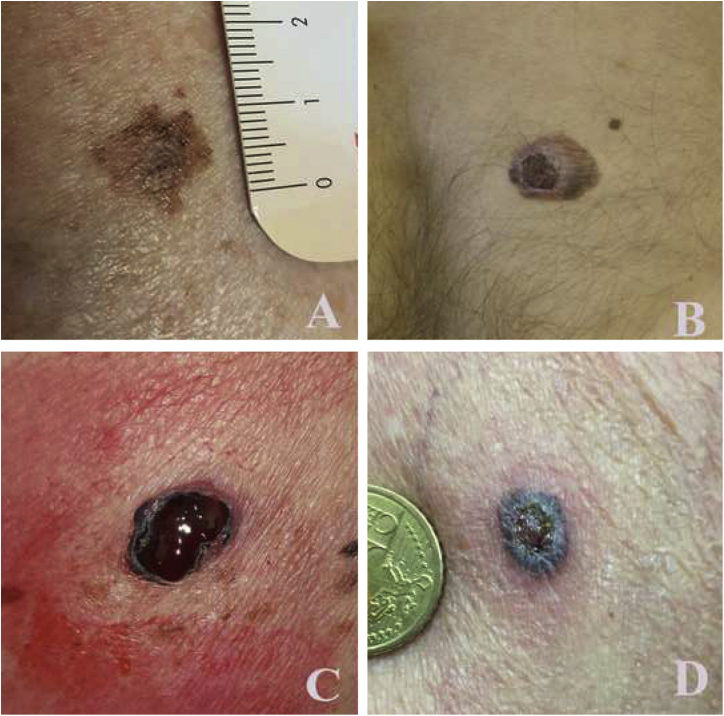
Examples of lesions and image quality acquired with mobile phones and sent to galliera.telederm.it: (A),83-year-old man, back: T1a melanoma; (B), 36-year-old man, back: T2b melanoma; (C), 68-year-old women, left flank: T3b melanoma; (D), 72-year-old man, left arm: pigmented basal cell carcinoma.

The present pilot experience suggests that a specific Primary Care Physicians’ dermatology and TD personal interactive training in small groups reduces the number of teleconsultations for benign skin lesions while the pick-up rate for skin cancers consistently improves resulting in early diagnosis of melanoma and NMSC and better care for high need dermatological patients (i.e., severe psoriasis or severe acne). Considering also the increasing incidence of dermatological conditions with aging and the difficulties in reaching medical facilities particularly now during COVID-19 pandemic, this approach might result particularly interesting for geriatric patients.^5^, ^6^ Moreover, training in TD also improves image quality that does not represent a weakness of TD anymore. The low number of patients enrolled, and the monocentric study protocol represents limitations of the present study. Larger studies are needed to confirm the authors’ hypothesis.

## Financial support

This project was sustained by voluntaries contributions by Difa Cooper SpA, Almirall SpA and Unifarco SpA.

## Authors' contributions

Cesare Massone: Data collection, analysis, and interpretation; effective participation in research orientation; preparation and writing of the manuscript; study conception and planning; manuscript critical review.

Sanja Javor: Data collection, analysis, and interpretation; participation in research orientation; preparation and writing of the manuscript

Ilaria Amato: Data collection, analysis, and interpretation; effective participation in research orientation; critical literature review; preparation and writing of the manuscript.

Giovanni Biondo: Data collection, analysis, and interpretation; effective participation in research orientation; critical literature review; preparation and writing of the manuscript.

Alexandra Maria Giovanna Brunasso: Data collection, analysis, and interpretation; effective participation in research orientation; statistical analysis.

Rainer Hofmann-Wellenhof: Study conception and planning; manuscript critical review; intellectual participation in propaedeutic and/or therapeutic management of studied cases.

## Conflicts of interest

None declared.
